# Adult onset cryopyrin-associated periodic syndrome due to germline missense mutation in *NLRP3* in a previously healthy middle-aged woman

**DOI:** 10.3389/fimmu.2025.1652177

**Published:** 2025-08-25

**Authors:** Sung Ik Cho, Sheehyun Kim, Ju Yeon Kim, Jeong-Won Kim, Sujin Pyeon, Won-Woo Lee, Jin Kyun Park

**Affiliations:** ^1^ Division of Rheumatology, Department of Internal Medicine, Seoul National University Hospital, Seoul, Republic of Korea; ^2^ Department of Genomic Medicine, Seoul National University Hospital, Seoul, Republic of Korea; ^3^ Division of Rheumatology, Department of Internal Medicine, Chung-Ang University Gwangmyeong Hospital, Gwangmyeong, Gyeonggi, Republic of Korea; ^4^ Laboratory of Autoimmunity and Inflammation (LAI), Department of Biomedical Sciences, Seoul National University College of Medicine, Seoul, Republic of Korea; ^5^ Department of Microbiology and Immunology, Seoul National University College of Medicine, Seoul, Republic of Korea

**Keywords:** autoinflammatory diseases, cryopyrin-associated periodic syndrome (CAPS), anakinra, adult onset, mutation, IL-1β

## Abstract

**Background:**

Cryopyrin-associated periodic syndrome (CAPS) is an autoinflammatory disease caused by a gain-of-function mutation in the *NLRP3* gene, which regulates inflammasome-mediated interleukin-1β (IL-1β) production. This leads to recurrent episodes of fever, rash, and arthritis, typically beginning in childhood.

**Objective:**

To demonstrate the role of a missense mutation, c.386A>G, in *NLRP3* in adult-onset CAPS in a previously healthy middle-aged woman.

**Methods:**

Whole-exome sequencing was performed. Serum levels of IL-1β, interleukin-1 receptor antagonist (IL-1RA), and tumor necrosis factor α (TNF-α) were measured. CD14-positive monocytes, isolated from the patient before and during IL-1 inhibition therapy, were stimulated with lipopolysaccharide (LPS), and cytokine production was assessed.

**Results:**

A 47-year-old woman presented with recurrent periorbital swelling and inflammatory symptoms, along with elevated IL-1RA and IL-6 levels. Genetic analysis revealed a heterozygous missense mutation in the *NLRP3* gene (NM_001243133, c.386A>G, p.Lys129Arg). Serum IL-1RA levels were significantly elevated during active disease. Monocytes from the patient produced high levels of IL-1β and TNF-α upon LPS stimulation. Treatment with anakinra ameliorated all symptoms and normalized inflammatory cytokine overproduction in the monocytes.

**Conclusion:**

We report a case of adult-onset CAPS in a previously healthy woman, caused by a missense mutation (c.386A>G) in the *NLRP3* gene, who exhibited a remarkable response to anakinra treatment. Autoinflammatory diseases should be considered in patients presenting with fever, skin rashes, and systemic symptoms, regardless of age.

## Introduction

Autoinflammatory diseases are characterized by abnormal activation of inflammatory pathways in the absence of antigen-driven autoimmunity (1). Dysregulated inflammasome activation leads to excessive production of interleukin-1β (IL-1β), resulting in recurrent sterile inflammation that manifests as episodic fever, rashes, and arthritis, among other symptoms (2). Gain-of-function (GOF) variants in the *NLRP3* gene cause excessive activation of the *NLRP3* inflammasome, leading to IL-1β overproduction (3). The excellent clinical response to IL-1β-targeted therapies underscores its critical role in disease pathogenesis (4).

Heterozygous missense mutations in *NLRP3* cause autosomal dominant cryopyrin-associated periodic syndromes (CAPS) (5). The three major subtypes of CAPS—familial cold autoinflammatory syndrome (FCAS), Muckle–Wells syndrome (MWS), and neonatal-onset multisystem inflammatory disorder (NOMID)—typically present early in life. In contrast, somatic *NLRP3* mutations with mosaicism, restricted to myeloid cell lines, have been associated with adult-onset CAPS (6). However, reports of adult-onset CAPS due to a heterozygous germline mutation are rare (7). Mutations in *NLRP3* can be clinically insignificant or pathogenic, with variable penetrance depending on the specific mutation site (8). With the increasing availability of genetic testing, many variants previously classified as “variants of uncertain significance” are now being reclassified as pathogenic based on emerging clinical and functional evidence (9).

In this study, we identified a case of adult-onset CAPS associated with a germline missense mutation at c.386A>G (p.Lys129Arg) in the *NLRP3* (NM_001243133) gene in a previously healthy middle-aged Korean woman who presented with chronic, recurrent episodes of periorbital swelling, fever, rashes, and arthritis. The patient exhibited a dramatic response to anti–IL-1 treatment. This finding supports the pathogenicity of this variant in the Asian population and suggests that it may be an underrecognized cause of recurrent febrile illness.

## Methods

### Study subjects

Blood samples were collected from the patient and healthy controls without autoimmune diseases after written informed consent was obtained. This study was approved by the Institutional Review Board (IRB) of Seoul National University Hospital (IRB No. H-2306-123-1440).

### Genomic analysis

Genomic DNA was extracted from the patient’s peripheral blood and fragmented using a Covaris E220 focused ultrasonicator (Covaris, Woburn, MA, USA). Whole-exome capture and library preparation were performed using the Agilent SureSelect All Exon V8 kit and the SureSelectXT Target Enrichment Protocol (Agilent Technologies, Santa Clara, CA, USA). Whole-exome sequencing (WES) was conducted on the Illumina NovaSeq 6000 platform (Illumina, San Diego, CA, USA) using 150 base pair (bp) paired-end sequencing.

Sequencing reads were aligned to the reference human genome (GRCh37/hg19) using Burrows–Wheeler Aligner (BWA v0.7.17). Variant calling followed the GATK Best Practices pipeline (v4.0.2.1). Single-nucleotide variants (SNVs) and small insertions/deletions (indels) were identified using an in-house pipeline incorporating SNVer (v0.5.3) and LoFreq (v2.1.2). Variants were functionally annotated using SnpEff (v5.0e).

### Human monocyte stimulation and cytokine analysis

Peripheral blood mononuclear cells (PBMCs) were isolated using density gradient centrifugation with Ficoll-Separating Solution (BIOCHROM Inc., Cambridge, UK). Monocytes were subsequently isolated from PBMCs using anti-CD14 magnetic beads (Miltenyi Biotec Inc., Auburn, CA, USA).

Isolated monocytes were stimulated with lipopolysaccharide (LPS) at a concentration of 10 ng/mL for 24 h. Cytokine levels—including interleukin-1 receptor antagonist (IL-1RA), interleukin-1β (IL-1β), interleukin-6 (IL-6), and tumor necrosis factor α (TNF-α)—were quantified from plasma or culture supernatants using commercial human enzyme-linked immunosorbent assay (ELISA) kits (Thermo Fisher Scientific, Waltham, MA, USA). Optical density measurements were obtained using an Infinite M200 microplate reader (Tecan, Männedorf, Switzerland).

## Results

### Case presentation

**Figure 1:**
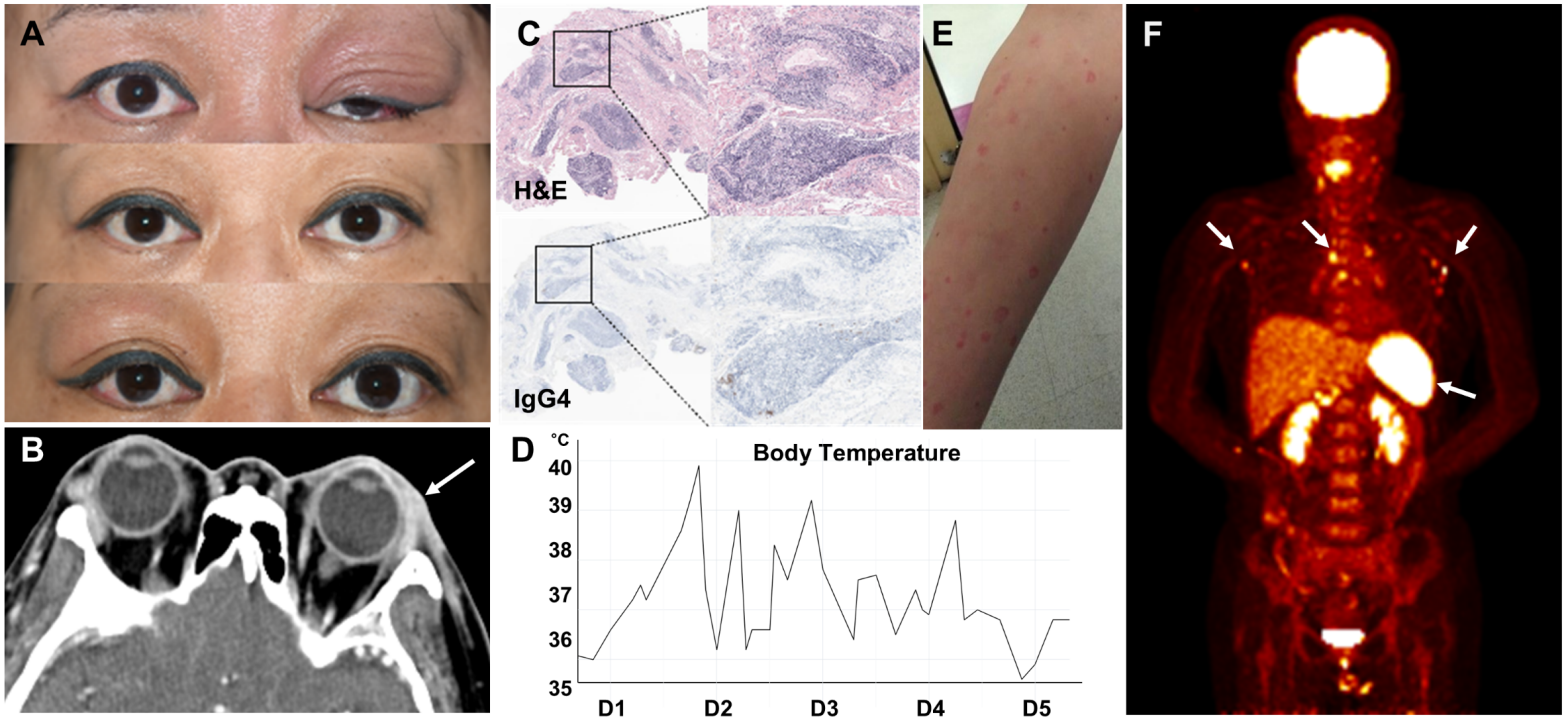
Recurrent periorbital swelling. **(A)** Recurrent and alternating swelling of the patient’s eyelids. **(B)** CT image of the orbital area showing soft tissue infiltration in the preseptal region, lacrimal gland, and left orbit (arrow). **(C)** Lacrimal gland biopsy showing multifocal lymphoplasmacytic infiltration (upper panel) and a few IgG4-positive cells (lower panel). **(D)** Temperature graph showing the patient’s fever pattern during the first 5 days of admission. **(E)** Maculopapular eruption. **(F)** PET scan showing increased metabolic activity in lymph nodes and spleen (arrows).

A 47-year-old Korean woman with hypothyroidism awoke with swelling in her left eyelid, which was red, warm, itchy, and tender to the touch. She also experienced a severe headache located around the eye and the left side of her head (**Figure 1A**). There was no known family history of autoimmune or autoinflammatory diseases, including recurrent febrile illnesses. A computed tomography (CT) scan of the head and neck revealed soft tissue infiltration in the preseptal region, lacrimal gland, and left orbit, along with enlarged cervical lymph nodes (**Figure 1B**). A biopsy of the affected eyelid showed multifocal lymphoplasmacytic infiltration with a mixed population of CD3^+^ T cells and CD20^+^ B cells, with a normal Ki-67 proliferation index. IgG4-producing cells were scarce (**Figure 1C**).

Anti-thyroid peroxidase and anti-thyroglobulin antibody levels were >4,000 IU/mL and 253 U/mL, respectively. Thyroid function test results were within normal limits. Suspecting an autoimmune disease such as thyroid-associated ophthalmopathy, she was treated with weekly methylprednisolone pulse therapy (250–500 mg) for 2 months, resulting in partial improvement. However, periorbital swelling recurred, alternating between the left and right eye over subsequent months.

Following her third dose of the COVID-19 vaccine, she developed fever with temperatures up to 40°C, cervical lymphadenopathy, skin rash, abdominal pain, myalgia, and arthralgia. The fever was intermittent (**Figure 1D**). The skin rashes were generalized, erythematous, confluent, maculopapular, and urticarial, measuring 1–2 cm in diameter. They were mildly pruritic and painful (**Figure 1E**). Laboratory results revealed a white blood cell (WBC) count of 9,230/µL, C-reactive protein (CRP) level of 9.20 mg/dL (normal <0.5 mg/dL), and erythrocyte sedimentation rate (ESR) of 91 mm/h. Her ferritin level was 2,341.4 ng/mL (normal: 21.8–274.7 ng/mL). Both serum and urine protein electrophoresis (PEP) showed no evidence of monoclonal gammopathy. Rheumatoid factor, antinuclear antibody (ANA), and anti-neutrophil cytoplasmic antibody (ANCA) were negative. An extensive infectious workup, including blood and urine cultures, was also negative.

CT imaging of the neck, chest, and abdomen showed diffuse lymphadenopathy and mild splenomegaly. A positron emission tomography (PET) scan revealed increased metabolic activity in the lymph nodes, bone marrow, and spleen (**Figure 1F**). A cervical lymph node biopsy demonstrated lymphoid follicular hyperplasia without evidence of malignancy. A bone marrow biopsy revealed reactive normocellular marrow without malignant cells.

**Figure 2:**
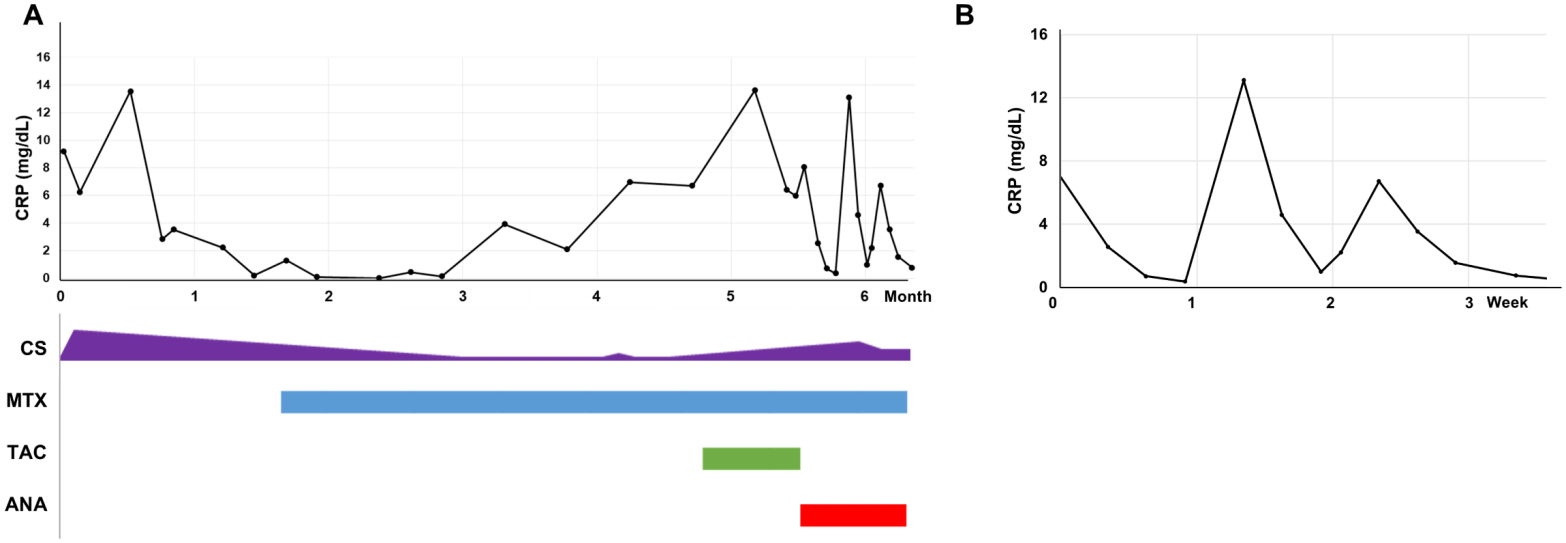
C-reactive protein levels in response to treatment. **(A)** C-reactive protein (CRP) levels over the first 6 months of treatment. **(B)** CRP changes with and without anakinra. ANA, anakinra; CRP, C-reactive protein; CS, corticosteroids; MTX, methotrexate; TAC, tacrolimus.

The fever persisted, with spikes up to 40°C. CRP and ferritin levels rose to 13.5 mg/dL and 16,567.19 ng/mL, respectively. Based on the constellation of fever, skin rash, diffuse lymphadenopathy, splenomegaly, and markedly elevated ferritin levels, adult-onset Still’s disease (AOSD) was suspected. Prednisolone 30 mg/day was initiated, with partial response. However, the patient continued to experience fever, fatigue, and recurrent periorbital inflammation, although CRP and ferritin levels declined to 2.82 mg/dL and 86.6 ng/mL, respectively. Fever returned when the prednisolone dose was tapered below 20 mg/day. Tocilizumab, methotrexate, and later tacrolimus provided little to no clinical improvement (**Figure 2A**).

An acquired autoinflammatory disease was suspected. A daily subcutaneous dose of anakinra (100 mg) was initiated. Remarkably, fever, constitutional symptoms, and periorbital edema resolved within hours of the first injection. For the first time since disease onset, the patient reported feeling completely well. CRP levels normalized to 0.35 mg/dL (normal <0.5 mg/dL) after 6 days of anakinra treatment. However, when anakinra was interrupted, both symptoms and CRP levels worsened immediately (**Figure 2B**).

### Genomic analysis

**Figure 3:**
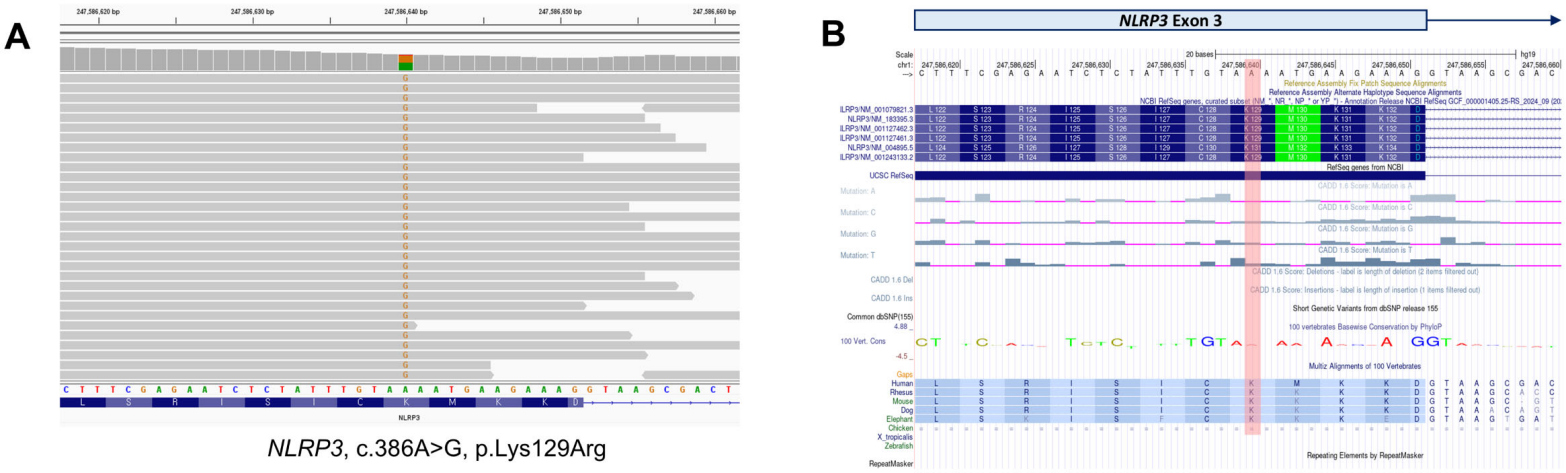
Genetic analysis. **(A)** Whole-exome sequencing identified a non-synonymous single-nucleotide variant in the NLRP3 gene. The variant was detected in 41 sequencing reads, with a variant allele frequency of 45.6%. **(B)** The NLRP3 variant results in the substitution of the 129th amino acid from lysine (K) to arginine (R) in exon 3 of transcript NM_001243133. The UCSC Genome Browser (https://genome.ucsc.edu/) was used to visualize the variant.

To identify the underlying genetic cause of the disease, next-generation sequencing-based whole-exome sequencing (WES) was performed. Among the autoinflammation-related genes, a heterozygous non-synonymous single-nucleotide variant (SNV) in the *NLRP3* gene (NM_001243133, c.386A>G, p.Lys129Arg) was identified and suspected to be the causative pathogenic change. Of the 90 total sequencing reads at this position, 41 (45.6%) carried the variant allele, consistent with a heterozygous germline mutation (**Figure 3**).

### Plasma cytokines analysis

**Figure 4:**
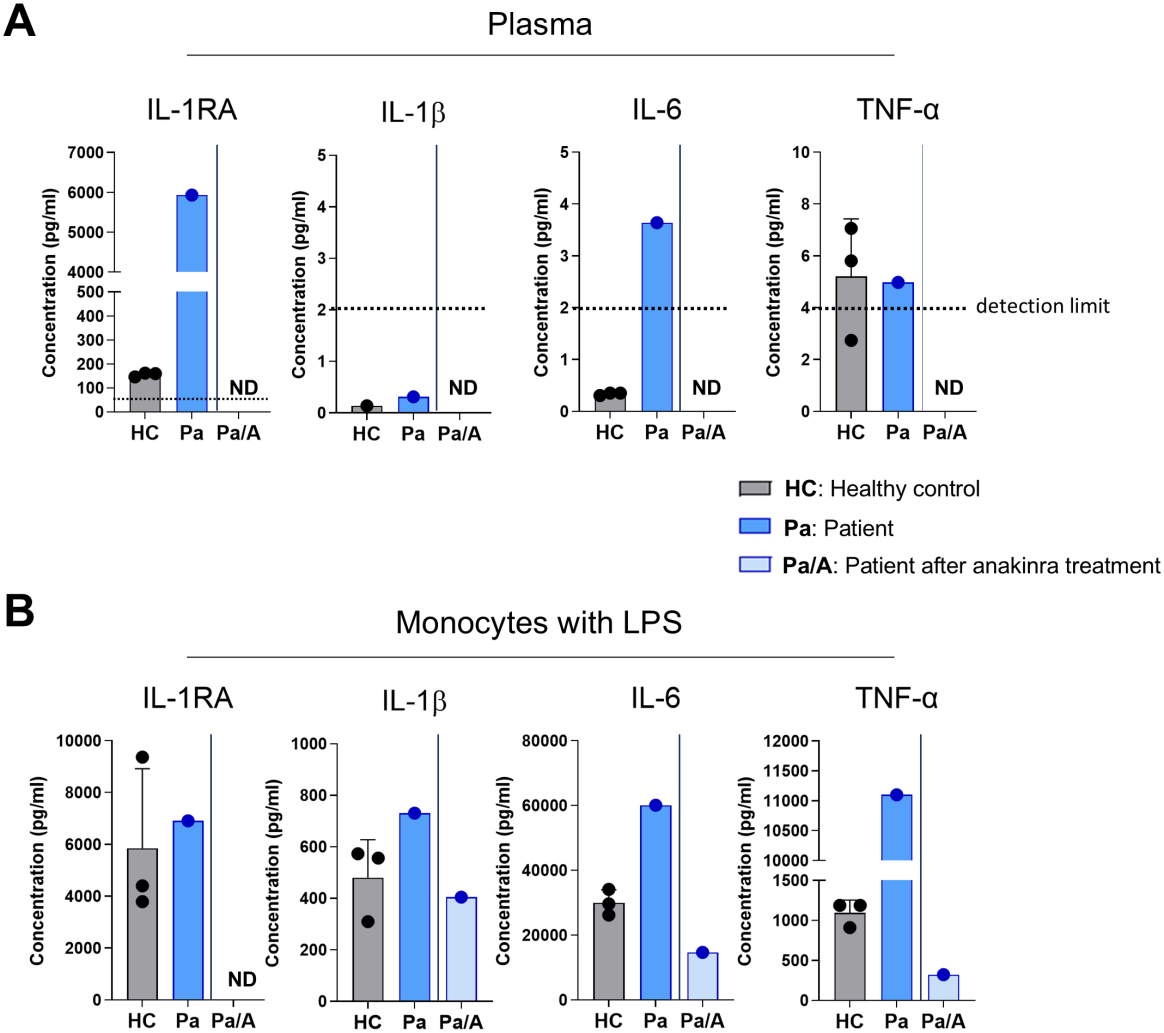
Elevated proinflammatory cytokine levels and hyperinflammatory response upon stimulation. **(A)** Cytokine concentrations in the plasma of healthy controls (n = 3) and the patient, before and after anakinra treatment, measured using ELISA. The dotted line represents the detection limit. **(B)** Purified CD14^+^ monocytes were stimulated with lipopolysaccharide (LPS, 10 ng/mL) for 24 h, and cytokine levels in the supernatant were quantified by ELISA. LPS, lipopolysaccharide; ND, not detected; Pt, patient.

The patient’s plasma interleukin-1 receptor antagonist (IL-1RA) level was markedly elevated at 5,933 pg/mL, compared with 155.9 ± 8.9 pg/mL in three healthy controls (**Figure 4A**). IL-1β levels were low in both patient and controls, while IL-6 level was slightly elevated in the patient. TNF-αlevels were comparable to those in controls. Following treatment with anakinra, plasma levels of IL-1RA, IL-1β, and IL-6 became undetectable.

### Cytokine production upon stimulation

Upon LPS stimulation, CD14^+^ monocytes from the patient produced IL-1RA levels comparable to controls (6,914 pg/mL vs. 5,856 ± 3,060 pg/mL). However, the patient’s monocytes secreted higher levels of IL-1β (730.6 pg/mL vs. 479.7 ± 147.7 pg/mL) and IL-6 (60,195 pg/mL vs. 30,029 ± 3,973 pg/mL) than healthy controls. TNF-α production was approximately 10-fold higher in the patient’s monocytes (11,102 pg/mL vs. 1,095 ± 160 pg/mL). Monocytes isolated after anakinra treatment produced lower levels of IL-1RA, IL-1β, and IL-6—even compared to controls (**Figure 4B**).

## Discussion

In this report, we describe a case of adult-onset CAPS in a previously healthy middle-aged Korean woman who developed recurrent high fevers, skin rashes, eyelid swelling, myalgia, and arthralgia, accompanied by elevated plasma IL-1RA and IL-6 levels. Genetic analysis revealed a heterozygous missense mutation in the *NLRP3* gene, and *in vitro* studies demonstrated increased production of the proinflammatory cytokines IL-1β and IL-6 upon stimulation. The patient responded remarkably to treatment with an IL-1β inhibitor.

The constellation of recurrent fevers, urticarial rash, arthritis, and myalgia raised suspicion for an autoinflammatory disease, including CAPS (1). CAPS results from gain-of-function mutations in *NLRP3*, leading to aberrant inflammasome activation and excessive IL-1β production (4). CAPS typically manifests early in life, often within the first few years. Therefore, given the new onset of febrile disease in this middle-aged patient, alternative diagnoses such as adult-onset Still’s disease (AOSD), acquired autoinflammatory conditions like VEXAS syndrome, or myelodysplastic syndrome with secondary autoimmunity were initially considered (10, 11). However, the patient’s suboptimal response to glucocorticoids and IL-6 inhibitors, along with a negative cancer workup, made AOSD and hematologic malignancies less likely (12). These unusual findings, including the episodic nature of inflammation, led to suspicion of an autoinflammatory disease. The diagnosis of adult-onset CAPS was confirmed by identifying a heterozygous missense mutation in *NLRP3* (rs188623199).

The patient’s initial presentation with eyelid swelling—fluctuating in severity and occurring in the absence of systemic inflammatory signs—led to a misdiagnosis of thyroid-associated ophthalmopathy, for which corticosteroid therapy provided only partial improvement. However, the subsequent emergence of systemic features, such as episodic fever and rash, prompted consideration of an autoinflammatory etiology. The rapid resolution of all symptoms, including eyelid swelling, following IL-1 blockade further supported this possibility and led to genetic testing for autoinflammatory diseases. This highlights how atypical features of adult-onset CAPS can delay diagnosis.

The identified *NLRP3* variant (NM001243133, c.386A>G, p.Lys129Arg), rs188623199, was classified as a variant of uncertain significance in the ClinVar database (variant ID: 234287). According to the Genome Aggregation Database (gnomAD), its allele frequency (AF) was 0.003%, with a higher AF of 0.1% in the East Asian population. Although several in silico prediction tools classify this variant as likely benign (CADD score: 13.7; REVEL: 0.0480; SpliceAI: 0.00; phyloP: 0.669), it has been reported in patients with *NLRP3*-associated autoinflammatory diseases, particularly among Chinese and Japanese populations (13–15).

More specifically, the p.Lys129Arg variant was recently reported as potentially pathogenic in a cohort of 16 Chinese patients with adult-onset NLRP3-associated autoinflammatory disease (13). The patient carrying this variant presented with fever, myalgia, and lymphadenopathy, similar to our case, except for the presence of periorbital swelling. Both patients lacked sensorineural hearing loss, a common feature of classic CAPS (16).

Feng et al. analyzed 534 NLRP3 variants, including many previously classified as variants of uncertain significance, using data from INFEVERS and ClinVar (17). Their findings suggest that certain amino acid substitutions may stabilize the active NLRP3 conformation, disrupt its autoinhibited state, and promote oligomerization, contributing to variable inflammasome activation. This may explain the wide range of clinical phenotypes associated with NLRP3 variants.

Our findings support that p.Lys129Arg may be a pathogenic driver and should be considered likely pathogenic, particularly in East Asian populations. This case highlights the disconnect between in silico predictions and clinical outcomes and underscores the importance of interpreting rare variants within the appropriate clinical context.

Adult-onset autoinflammatory diseases are rare and exhibit variable clinical manifestations, influenced by both regulatory mechanisms and the mutation-specific effects of *NLRP3* variants (18, 19). This patient initially presented with localized periorbital inflammation, which progressed to systemic disease following her third dose of the COVID-19 vaccine—a known trigger for dysregulated *NLRP3* activation (20). *NLRP3* variants with reduced penetrance may require an additional inflammatory trigger, or “second hit,” such as a viral infection or vaccination, to precipitate overt clinical disease (21).

Before treatment with an IL-1 inhibitor, plasma levels of IL-1RA and IL-6 were elevated. Treatment with the IL-1 inhibitor anakinra normalized these levels, consistent with previous reports (22). Interestingly, IL-1β and tumor necrosis factor α (TNF-α) levels were not elevated. It remains unclear whether IL-1β production is locally elevated within inflamed tissues. Alternatively, excess IL-1RA may have neutralized circulating IL-1β, rendering it undetectable in serum. Furthermore, the limited sensitivity of ELISA for IL-1β in plasma may also account for the low measured levels.

Notably, monocytes isolated from the patient during active disease exhibited increased production of IL-1β, IL-6, and TNF-α upon stimulation compared with healthy controls. IL-1β is known to potentiate IL-6 and TNF-α production via NF-κB activation, which may have contributed to the heightened inflammatory response (23). Following anakinra treatment, the hyperinflammatory response was normalized. Further studies are needed to confirm whether the observed *NLRP3* variant directly enhances inflammasome activity *in vivo*.

The remarkable response to IL-1β inhibition observed in this patient is characteristic of CAPS. In contrast, her poor response to IL-6 inhibitors and the suboptimal efficacy of corticosteroids is consistent with reports indicating that CAPS is primarily an IL-1β–driven disease (24). This underscores IL-1β as the central pathogenic cytokine, while IL-6 elevation may be secondary. In patients with suspected adult-onset Still’s disease (AOSD), an inadequate response to IL-6 inhibition should prompt further evaluation for inflammasome-mediated disease.

## Conclusions

In conclusion, we report a case of adult-onset CAPS in a previously healthy woman, driven by a missense mutation in the *NLRP3* gene (c.386A>G, p.Lys129Arg). The patient demonstrated a dramatic response to anakinra, highlighting the importance of genetic analysis in the diagnosis of autoinflammatory diseases. This case underscores the need to consider monogenic autoinflammatory diseases in patients presenting with fevers, skin rashes, and systemic symptoms—particularly when conventional therapies fail.

## Data Availability

The raw data supporting the conclusions of this article will be made available by the authors, without undue reservation.
